# Safe Drinking Water and Its Impact on Children’s Growth and Development: A Systematic Review

**DOI:** 10.3390/ijerph23030313

**Published:** 2026-03-02

**Authors:** Tria Rosemiarti, Diana Sunardi, Netta Meridianti Putri

**Affiliations:** 1 Department of Nutrition and Science, The Tirta Investama, Jakarta 12950, Indonesia; 2 Department of Nutrition, Faculty of Medicine, Universitas Indonesia–Dr. Cipto Mangunkusumo General Hospital, Jakarta 10430, Indonesia; diana_sunardi@yahoo.com; 3 Department of Nutrition, Universitas Tidar, Magelang 56116, Indonesia; nettamp@gmail.com

**Keywords:** drinking water quality, child growth, cognitive development

## Abstract

**Highlights:**

**Public Health Relevance—How does this work relate to a public health issue?**

- Microbiological contamination of drinking water, particularly Escherichia coli, is strongly associated with stunted growth and reduced height-for-age Z-scores in children aged 0–5 years, directly contributing to the global burden of child undernutrition that affects an estimated 149 million children.
- This systematic review synthesizes evidence from low- and middle-income countries (LMICs) where inadequate WASH infrastructure amplifies children’s vulnerability to contaminated drinking water, underscoring the urgent public health need for safe water access as a foundation of child health and development.

**Public Health Significance—Why is this work of significance to public health?**

- This review provides a comprehensive synthesis of the evidence linking drinking water quality to both physical growth and cognitive development in early childhood, demonstrating that contaminated water not only causes stunting but may also impair neurodevelopmental outcomes—a dual burden with lifelong implications for educational attainment and economic productivity.
- The findings highlight that single-component water treatment interventions (e.g., chlorination alone) are insufficient to substantially improve child growth outcomes; integrated WASH-nutrition strategies addressing multiple exposure pathways are required, which has significant implications for the design and funding of public health programs aligned with Sustainable Development Goal (SDG) 6.

**Public Health Implications—What are the key implications or messages for practitioners, policy makers and/or researchers in public health?**

- Policy makers and program implementers should prioritize integrated WASH and nutrition interventions over water-quality-only approaches, as combined strategies targeting water, sanitation, hygiene, and nutritional support have demonstrated greater benefits for child growth and cognitive outcomes across diverse LMIC settings.
- Researchers should address critical gaps in this field, including the limited evidence on chemical contaminants’ effects on child development, the need for longitudinal studies with standardized cognitive outcome measures, and the importance of conducting well-registered, prospective studies in diverse geographic settings to strengthen evidence for global policy translation.

**Abstract:**

Access to safe drinking water is critical for child growth and development. However, microbial contamination is a constant threat in many low- and middle-income countries. The current systematic review sets out to examine the evidence of drinking water quality and the physical and cognitive development of children aged 0 to 5 years. The review authors conducted a comprehensive search of SCOPUS, EBSCO, PubMed, the Cochrane Library, and Google Search for cohort studies and clinical trials conducted in English between the years 2010 and 2025. Of 222 studies, 15 were included in the review and the majority were conducted in low- and middle-income countries The findings consistently demonstrate that microbiological contamination, predominantly by *Escherichia coli* (the primary water quality indicator examined across studies), is associated with an increased risk of stunting (odds ratio up to 4.14) and reductions in height-for-age Z-scores (HAZ) (by 0.29–0.57). There is currently limited evidence in the studies reviewed that suggests a correlation between the presence of unsafe drinking water and a decrease in cognitive development; however, the evidence is insufficient and warrants further study. Integrated water, sanitation and hygiene (WASH) and nutrition programs had promising growth results, which varied depending on the initial sanitation coverage of the target population, adherence to the intervention, and the overall design of the program. To sum up, contaminated drinking water negatively affects physical and cognitive development during early childhood. Comprehensive WASH–nutrition strategies need to be implemented to reduce this impact and further progress towards Sustainable Development Goal (SDG) 6.

## 1. Introduction

In this review, we primarily focus on microbiological water quality, the presence or absence of fecal indicator bacteria, particularly *Escherichia coli* (*E. coli*), as this represents the predominant contaminant type examined in available studies on child growth and development. While chemical contaminants such as arsenic, nitrates, and fluoride are acknowledged as important risks to child health [1], the current evidence base on drinking water and child development is heavily weighted toward microbiological contamination, with limited data on chemical contaminants’ specific effects on growth outcomes. This distinction is important for interpreting our findings and identifying areas requiring further research.

Access to safe drinking water is a critical determinant of child health, particularly for growth and development in early life. The World Health Organization (WHO) estimates that contaminated drinking water contributes to over 500,000 deaths annually from diarrheal diseases, with children under five years being the most vulnerable [2]. Poor water quality, characterized by microbial pathogens such as *Escherichia coli* or chemical contaminants like arsenic and nitrates, poses significant risks to children, leading to adverse outcomes such as stunting, wasting, and impaired cognitive development [3,4]. These risks are amplified in low- and middle-income countries (LMICs), where inadequate water, sanitation, and hygiene (WASH) infrastructure exacerbates health challenges [5,6].

Early childhood is a critical period during which exposure to contaminated water can disrupt physical and cognitive development through environmental enteric dysfunction. Microbial contamination in drinking water is linked to environmental enteric dysfunction (EED), a subclinical condition that impairs nutrient absorption, increases systemic inflammation, and contributes to stunting and reduced linear growth [7]. For example, chronic exposure to fecal pathogens in water is associated with a higher risk of diarrheal diseases and helminth infections, which further compromise nutritional status and growth [8]. Additionally, poor water quality can hinder cognitive development by increasing infection-related inflammation, which affects neurodevelopmental processes during critical growth windows [9]. The global burden of child undernutrition underscores the urgency of addressing water quality, with approximately 149 million children under five classified as stunted and 45 million as wasted in 2020 [10].

Beyond acute diarrheal diseases, contaminated drinking water contributes to a range of severe health impacts in children. Chemical contaminants in water, including arsenic, nitrates, and fluoride, pose particular risks to young children who consume more water per kilogram of body weight compared to adults [1]. Chronic microbial exposure leads to environmental enteric dysfunction (EED), a subclinical condition characterized by intestinal inflammation and impaired nutrient absorption [6,7,8]. This condition contributes significantly to growth faltering independent of clinical diarrhea episodes [10]. Furthermore, emerging evidence suggests that poor water quality during early childhood may affect neurodevelopmental outcomes, with potential long-term consequences for cognitive function and educational achievement [11,12,13].

Despite the recognized link between water quality and child health, systematic reviews have primarily focused on WASH interventions broadly, with limited attention to the specific role of drinking water quality in child growth and development [11,14]. This gap is critical, as stunting and cognitive deficits in early childhood have lifelong implications, including reduced educational attainment and economic productivity [4]. Addressing this knowledge gap is essential for informing targeted interventions and policies, particularly in LMICs, where access to safe water remains a challenge. This systematic review aims to synthesize evidence from prospective cohort studies and clinical trials to evaluate the impact of drinking water quality on child growth and development, focusing on anthropometric outcomes, such as stunting, height-for-age Z-scores (HAZ) and cognitive development, such as memory and language skills. By examining studies conducted over the past 15 years, this review seeks to identify mechanisms linking water quality to child health and propose interventions to mitigate adverse effects, aligning with Sustainable Development Goal (SDG) 6 for clean water and sanitation [15].

## 2. Materials and Methods

### 2.1. Study Design

**Figure 1:**
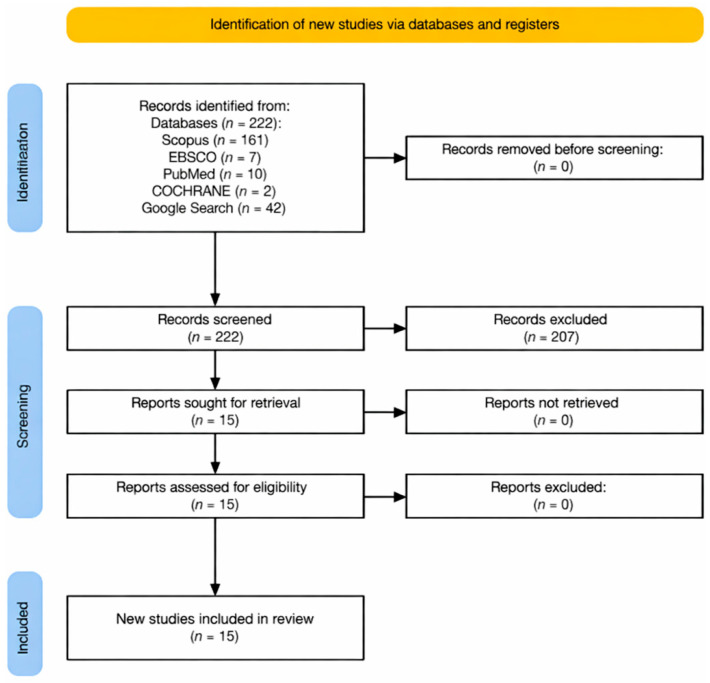
PRISMA flow diagram for study selection process.

This systematic review assesses the impact of drinking water quality on child growth and development. The study adhered to the Preferred Reporting Items for Systematic Reviews and Meta-analysis (PRISMA) guidelines to ensure methodological rigor and transparency [16]. The completed PRISMA 2020 checklist is provided as Supplementary File S1. The review was conducted without prior registration in a systematic review registry. A systematic quantitative approach was employed, synthesizing numerical data from prospective cohort studies and clinical trials published between January 2010 and May 2025. The review was guided by a conceptual framework (Figure 1) addressing the research question: How does the quality of drinking water influence child growth and development?

### 2.2. Eligibility Criteria

Two sets of defined criteria were used to determine the relevance, rigor, and comparability of the studies. Inclusion criterion one (IC1) relates to the study details, focusing on primary, peer-reviewed, and published articles written in English between the years 2010 and 2025. All studies had to analyze the relationship of drinking water quality on the growth or developmental outcomes of children. Inclusion criterion two (IC2) relates to the study population and study design. Articles were only eligible if the participants were children aged 0–5 years who had no comorbid medical conditions, and studies were designed as either prospective cohort studies, randomized clinical trials, cross-sectional studies, or case–control studies. Even though prospective studies were prioritized, cross-sectional and case–control studies were included if they contained pertinent quantitative data on water quality and child development outcomes. In addition to this, studies had to include one or more quantitative measures as an embodiment of development or growth, such as height-for-age Z-scores (HAZ), stunting prevalence, or scores from standardized cognitive tests. Although the exclusion of studies that measured environmental enteric dysfunction (EED) biomarkers, within the provided studies, EED serves as an interpretative framework to describe the relationship between quality of water and growth outcomes through the relevant biomarkers.

Studies were excluded if they had non-human participants, if they were in a language other than English, if they were not quantitative with respect to child growth outcomes, and if they did not measure the quality of drinking water. While the scope of the review included both types of contaminants, as mentioned in the introduction, the studies that were included focused mainly on the assessment of microbial contaminants (especially *E. coli* and fecal coliforms), because evidence is more readily available in this area. Chemical contaminants were included to the extent that data were available. These criteria ensured that the studies included were relevant to the review’s focus on child growth and development and of high quality.

### 2.3. Information Sources and Search Strategy

A comprehensive literature search was conducted across five databases: SCOPUS, EBSCO, PubMed, Cochrane Library, and Google Search. Search terms included “drinking water quality,” “water quality,” “contamination,” “microbial contamination,” “*E. coli*,” “*Escherichia coli*,” “child growth,” “child development,” “cognitive development,” “stunting,” and “anthropometric outcomes,” combined with medical subject headings (MeSH) for water quality and child health. Boolean operators (AND, OR) were used to refine the searches (Table 1). The search was restricted to publications from 2010 to 2025 to capture recent evidence. Reference lists of included studies were manually reviewed to identify additional relevant articles.

### 2.4. Study Selection Process

We streamlined the study selection process into four phases per Rethlefsen et al. [17]. In the first phase, we retrieved 222 records from Scopus, EBSCO, PubMed, Cochrane Library, and Google Search. In the second phase, we screened titles and abstracts per the first inclusion criterion (IC1). At this stage, 207 records were removed due to irrelevance, duplication, or failure to satisfy IC1.

The next phase involved screening for the full-text eligibility of the remaining 15 articles based on inclusion criterion 2 (IC2). All 15 studies satisfactorily met IC2 and were thus sampled for the final qualitative synthesis. A further reference list screening of the eligible studies was conducted, and additional studies were found to be noncompliant with the inclusion criteria.

Figure 1 summarizes the entire process using the PRISMA flow diagram. The authors screened and assessed the studies independently, and any disagreements that arose were discussed and resolved. A uniform data extraction template was filled out to generate a summary table, and study attributes such as journal, year, setting, participants, methods, outcomes, and conclusions were incorporated.

### 2.5. Data Collection and Quality Assessment

Using a standardized extraction form, the study design, sample size, demographics, drinking water quality, anthropometric and cognitive outcomes, and key findings with effect sizes and confidence intervals were recorded.

Two established instruments were used for the quality assessment. The Newcastle–Ottawa scale (NOS) was used to assess the methodological quality of the cohort studies focusing on selection, comparability, and outcome assessment [18]. The Cochrane risk of bias tool was used for clinical trials focusing on randomization, allocation concealment, and blinding [19]. The authors worked independently, and any discrepancies were resolved through discussion. Substantial heterogeneity among studies in terms of water quality measurement (e.g., *E. coli*, total coliform, presence/absence), outcome (stunting cut-offs, cognitive assessment), study design (cohort, RCT, cross-sectional) and population (age, nutrition status) impeded quantitative meta-analysis. There was no meaningful statistical pooling of the effect estimates.

## 3. Results

Out of the 222 articles found with the keywords provided, 15 met the inclusion criteria of being prospective cohort studies and clinical trials published between 2010 and 2025 on the relationship between drinking water quality during pregnancy and child growth and development outcomes (Table 2). The studies took place in low- and middle-income countries (LMICs) and were from Indonesia, Bangladesh, India, Uganda, Kenya and Mali, with sample sizes ranging from 84 to 8246. Their outcomes were measured in terms of stunting, HAZ, weight-for-age Z-score [WAZ], and memory, language, and numeracy test scores. Because of the varied study designs, water quality measurements, and outcomes, the data was narratively synthesized.

### 3.1. Anthropometric Outcomes

Out of ten studies that examined anthropometric measurements, eight studies found that poor quality of drinking water was related to negative child growth outcomes, specifically stunting. Haque et al. [25] found that the risk of stunting in Bangladeshi children aged 24 to 60 months increased by 9% (*p* < 0.01) due to high levels of *E. coli* contamination in drinking water. Unsafe water (E. coli positive) was associated with a lower height-for-age Z (HAZ) score of β = 0.29 (95% CI: 0.00–0.58), and stunting was 1.68 to 1.70 times more likely in Ugandan children aged 12 to 16 months, according to Lauer et al. [24]. Hasanah et al. [23] found that proper sanitation, usually associated with water quality, decreased the odds of stunting in Indonesian children (OR = 0.65, *p* < 0.01). Microbiologically unsafe water quality increased the stunting risk over four times (OR = 4.14, 95% CI: 1.65–10.44), as confirmed by Syaputri et al. [31], in children aged 6 to 59 months.

The interventions focused on water quality yielded mixed outcomes. While Null et al. [26] and Luby et al. [27] noted no considerable improvements in growth from water treatment alone (i.e., chlorination), nutrition-sensitive interventions did enhance HAZ (mean difference: 0.13–0.25, 95% CI: 0.01–0.36). Following a community-led sanitation intervention that indirectly improved water quality, Pickering et al. [22] noted a 0.18 increase in HAZ and a 6% decrease in stunting in Malian children under 2 years.

### 3.2. Cognitive Development Outcomes

Three out of five studies showed that cognitive development has a significant link with the quality of water. Wulan et al. [21] showed that in Indonesia, children whose mothers possessed safe water during pregnancy had better word list memory scores (95% CI: 0.01–0.19, *p* < 0.02) when they turned 9–12 years old. Cameron et al. [29] stated that in Indonesia, water accessibility reduced cognitive score deficits by 3–4.5% (*p* < 0.05) in children, though the presence of sanitation had a more significant impact. Pakhtigian [30] reported that in adolescents, improved water accessibility in early life increased scores in math (2–4%), reading (3–5%), and the Peabody picture vocabulary test (PPVT) (2–3%) across Ethiopia, India, Peru, and Vietnam, with the effects more pronounced in girls (*p* < 0.05). However, in India, adverse effects indicated a probability of groundwater pollution (e.g., fluoride).

### 3.3. Study Quality and Heterogeneity

We used the NOS for cohort studies and the Cochrane tool for clinical trials [18,19] to evaluate the studies’ quality. Most studies had a quality rating of moderate. Shortcomings were attributed to the lack of blinding, variable adherence to the intervention, and the use of proxy measures for water quality (e.g., *E. coli*). The lack of uniformity was due to the different study environments, types of contaminants (microbial or chemical), and different measurements of the outcomes. The evidence showed that of all the contaminants of drinking water, microbes were most often responsible for the poor growth and/or cognitive development of children. In most cases, chemical contaminants did not have the same effect.

## 4. Discussion

This systematic review details how the quality of drinking water, especially during microbial contamination in pregnancy and early childhood, impacts the physical and cognitive development of children. The studies conducted in Bangladesh, Uganda, and Indonesia [23,24,25,31] consistently demonstrate the link between E. coli water contamination and child stunting, pointing to EED as a key mechanism. Unsafe water, characterized by the presence of E. coli, was shown in Lauer et al. [24] to be linked to higher levels of EED, as measured by lactulose-mannitol ratios, which correlated with a 0.29 (95% CI: 0.00, 0.58) reduction in the length-for-age Z-score (LAZ) in Ugandan children. In the same way, Haque et al. [25] reported that children aged 24–60 months in Bangladesh exposed to water sources with a high level of E. coli contamination had a 9% (*p* < 0.01) greater risk of stunting. These studies support the hypotheses of Humphrey [6] regarding the adverse impacts of chronic exposure to fecal pathogens on the intestines caused by EED, weakening the intestines’ structural integrity, impairing the body’s ability to absorb nutrients, and causing a persistent inflammatory response that collectively promotes suboptimal child growth. Syaputri et al. [31] assigned a value to this risk when they reported that in Indonesian children, water that was microbiologically unfit for drinking increased the odds of stunting by a factor of 4.144 (95% CI: 1.65–10.44). Inadequate exposure to chemical contaminants, like nitrate and fluoride, contrasts with the situation regarding microbial contaminants.

**Figure 2:**
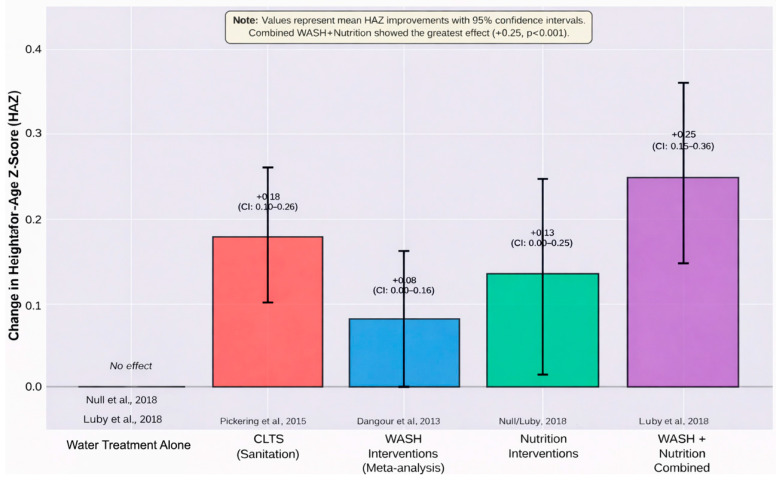
Comparative effectiveness of WASH and nutrition interventions on child growth. Combined WASH and nutrition interventions showed the greatest HAZ improvement (+0.25, 95% CI: 0.15–0.36). Error bars represent 95% confidence intervals [11,22,26,27].

Pakhtigian [30] noted negative academic achievement in India attributed to fluoride in groundwater and some anthropometric evidence. This discrepancy noted that microbial pollutants are associated with stunted growth, whereas chemical pollutants are linked to stunted growth accompanied by subtle and context-specific effects. Dangour et al. [11] noted that during the first two years of life, combined WASH interventions resulted in a statistically and clinically important increase in HAZ. Similarly, Freeman et al. [14] stated that better sanitation and the reduction of diarrheal disease have a protective effect on HAZ, although results for other anthropometric measures were inconsistent. These studies demonstrated that the level of child growth is clearly attributed to the level of WASH improvements. Figure 2 shows that WASH interventions combined with nutrition interventions produced the greatest HAZ improvements.

The interventions solely focusing on water quality, such as chlorination, showed little success in improving growth outcomes, as highlighted in large-scale studies conducted in Kenya and Bangladesh [26,27]. Null et al. [26] evaluated the effectiveness of water treatment on improving height-for-age Z-scores. Despite the seemingly high adherence rate of 45%, there were no noticeable improvements in height-for-age Z-scores, which could be attributed to the ongoing environmental contamination of the study areas with feces of farm animals or protozoan parasites such as Giardia and Cryptosporidium, which are not killed by routine chlorination. Luby et al. [27] described the same null effect on height-for-age Z-score improvement attributed to water interventions, although there was a 3.5% reduction in diarrhea among those receiving water, sanitation, and hygiene (WASH) interventions. The low improvement in water-only interventions might be attributed to the high baseline sanitation coverage in the study areas [26]. On the other hand, the synergistic effect of the combination of WASH and nutrition interventions was more pronounced. Luby et al. [27] reported that nutrition interventions (including lipid-based nutrient supplementation) resulted in an improvement of 0.25 (95% CI: 0.15–0.36) in height-for-age Z-scores, while the combination of WASH and nutrition interventions was associated with a reduction in the prevalence of severe stunting by 2.7%. Following a community-led total sanitation (CLTS) intervention in Mali, Pickering et al. [22] observed a 0.18 increase in height-for-age Z-scores and a 6% reduction in stunting among children aged two years and below, as water quality improved indirectly through a 23% reduction in open defecation among adults. The most effective improvement in water quality, combined with sanitation and nutritional support, targets multiple pathways of pathogen exposure and nutrient deficiency in high-risk environments.

The impacts on cognitive development outcomes provide further evidence concerning the lasting impact of the quality of water. Wulan et al. [21] demonstrated that children in Indonesia whose mothers had safe water while pregnant performed better on word list memory tests (95% CI: 0.01–0.19, *p* < 0.02) at ages 9–12, even after considering socioeconomic factors. Cameron et al. [29] stated that in Indonesia, the improved provision of water had the potential to lessen cognitive score deficits by 3–4.5%, although the provision of improved sanitation had a greater impact. Among the countries of Ethiopia, India, Peru, and Vietnam, Pakhtigian [30] noted that the early provision of improved water quality was associated with a 2–4% increase in adolescent math, a 3–5% increase in reading, and a 2–3% increase in PPVT scores, especially among females. The positive impacts on cognitive development may have been a result of the absence of pathogens, which leads to inflammation and better absorption of nutrients, especially during periods of rapid brain development. The findings concerning the girls in Pakhtigian’s study may be due to social reasons such as the greater responsibility of girls to fetch water, which may limit their opportunity to go to school in areas lacking safe water, or it may be due to biological reasons such as different responses of the immune system to infections.

Nonetheless, India’s negative cognitive impacts, as a result of possible fluoride exposure, emphasize the significance of region-specific water chemistry [30]. This indicates that while microbial contamination is the main cause of direct stunted growth, chemical pollutants may have a secondary impact on cognitive development, reinforcing the need for extensive assessments of water quality.

Policies founded on evidence from LMICs lacking SDG 6 defined ‘safely managed’ water services are of importance. In rural India, Johri et al. [28] reported a 7.4% (95% CI: -13.4% to −1.3%) decrease in underweight children after achieving SDG 6.1 standards (improved, pathogen-free water). Meanwhile, in the Lao People’s Democratic Republic, Som et al. [32] stated that 86.5% of household drinking water and 83.6% of water sources contained E. coli, which illustrates a disparity between water infrastructure and safety. The CLTS program in Mali is a scalable solution because of its community-level approach. It increased latrine coverage from 33% to 65% and subsequently decreased child diarrhea mortality by 54% [24]. At the community level, Syaputri et al. [31] cited the importance of household water treatment and hygiene, which, coupled with infrastructure, could reduce the risk of stunting. Hasanah et al. [23] showed that in the case of Indonesia, the stunting odds translate synergistically with the level of sanitation, i.e., with proper sanitation the stunting odds reduce by 0.645 (*p* < 0.01). The focus on changing behavior, ensuring adequate nutrition, and overcoming the vicious cycle of malnutrition and microbial contamination in high-pathogen environments is supported by integrated WASH-nutrition programs in the studies mentioned.

### 4.1. Understanding Context-Dependent Effectiveness

The systematic review conducted shows a clear pattern bringing together seemingly opposing findings from the literature. Three recent studies of good quality and randomized sampling—Clasen et al. in India [20], Null et al. in Kenya [26], and Luby et al. in Bangladesh [27]—despite having adequate sample sizes (>3500 children in all studies) and demonstrating high adherence (>70% in all studies) and a proven decrease in diarrheal disease, recorded no effect of any WASH (water, sanitation and hygiene) intervention on the growth of children. Out of the 8 studies in this review, the observational studies reported positive findings, while the rest were described as null. In our review, poor water quality was positively correlated with an increased risk of stunting.

Most studies end up being described as null due to perceived negative outcomes. Here, Null et al. [26] documented *E. coli* in over 70% of households, while 35% of households reported stunting, and Luby et al. [27] outlined the same findings in rural Bangladesh. Out of the other 3 null studies, this has the most significant impact on the rural demographic, with a feedback loop affecting children from several cross-contamination channels. Within the scope of the studies conducted, the provisions of the other 3 studies all provided household-based boosters.

The WASH Benefits trials themselves acknowledge this problem. Null et al. [26] remarked that even with the interventions, there was insufficient response to likely residual environmental contamination from animal excreta and deficient sanitation at the community level. Likewise, Luby et al. [27] noted that while nutrition interventions had a positive effect on linear growth (HAZ +0.25, 95% CI: 0.01–0.36), WASH interventions had no positive effect, implying that in these settings with high burdens, water treatment at the household level is but a fraction of the complete exposure that hinders child growth.

In contrast, in the study conducted by Pickering et al. in Mali [22], a community-led total sanitation (CLTS) methodology was used, which, among other things, addressed residual environmental contamination comprehensively. This led to a reduction of stunting by 6 percentage points and an improvement of 0.18 in HAZ. This community-based initiative achieved a 23% reduction of open defecation in adults and 43% in children, thereby modifying the broader exposure environment rather than replacing a single pathway. This implies that the extent of the impact of interventions is highly dependent on whether the methodology used can address the totality of environmental contamination in high-burden regions.

### 4.2. Critical Developmental Windows

Our studies identified specific age-related vulnerabilities, highlighting 6–24 months as a critical age period. Studies that documented poorer water quality and increased child stunting within this 6–24 month age range showed even stronger correlations. Lauer et al. [24] documented children between 12 and 16 months and noted that *E. coli*-contaminated water (unsafe water) resulted in 1.68–1.70 greater odds of stunting and lower length-for-age Z-scores (LAZ β = 0.29, 95% CI: 0.00–0.58). Joseph et al. [20] documented children in the 24–60 month range and noted a 9% greater chance of being stunted as a result of E. coli-contaminated water.

Water-based complementary feeding practices commence during the 6–24 month age range. During this period, children’s rapid growth and development occurs. Any stunting that occurs during this period is of significant concern because of the long-term consequences for the child. A disproportionate impact is expected from the implementation of interventions during this age range compared to the implementation of such interventions across the entire population.

### 4.3. Mechanistic Insights: Environmental Enteric Dysfunction

Lauer et al. explain how contaminated water hinders growth outcomes through environmental enteric dysfunction (EED). Using the EED biomarker lactulose:mannitol (L:M) ratios, the authors found that unsafe drinking water contributes to EED (β = −0.22, 95% CI: −0.44–0.00). Children with high EED markers had worsened LAZ scores and stunting, which depicts the cycle of exposure to pathogens through contaminated water, causing gut inflammation and negative nutrient absorption resulting in stunted growth.

The process of contaminated water → pathogen exposure → gut inflammation → nutrient malabsorption → stunted growth illustrates why the impacts of water quality remain even when there is no clinical diarrhea. EED has no clinical signs, meaning children can experience considerable stunting with no sickness, and this has been documented in numerous studies in the literature. This is pertinent to policy because it means there is no correlation between enteric pathogens and growth outcomes even when diarrhea is less prevalent (as in the null RCTs).

### 4.4. Long-Term Consequences of Cognitive Development

Since the majority of the studies we reviewed focused on physical development, we note that the studies by Wulan et al. and two other studies we review provide the first evidence of how the development of certain cognitive functions may affect children in the long term. Wulan et al. [21] show that in the range of 9–12 years, children whose mothers had access to safe water and sanitation during pregnancy had significantly better scores on the word list memory (WLM) test (95% CI: 0.01–0.19, *p* < 0.02). This finding suggests that exposure to water and sanitation during pregnancy has an impact that extends beyond the immediate period of pregnancy and may be seen in the years to come. Water and sanitation may have more far-reaching impacts rather than only immediate effects on children’s physical growth.

In developing a further understanding of the impact of modern sanitation on children, Cameron et al. [29] show that better sanitation in Indonesia results in a 4–5% reduction of stunting in children and a 4.5–7.5% improvement in cognitive test scores, with the greatest cognitive improvement observed in no-open-defecation communities. Considerable cognitive improvements occur in the absence of direct malnutrition affecting brain development, malnutrition of the important micro-nutrients, and potential neurotoxins from the bulk of the pollutants.

Pakhtigian [30] highlighted an interesting distinction between the impacts of microbial contamination and chemical contamination. The author attributed the negative impacts in India to possible groundwater contamination with arsenic or fluoride. By contrast, in Ethiopia, Peru, and Vietnam, access to improved water was associated with 2–4% better academic performance. This finding highlights the fact that within the SDG 6 indicators, “improved” water sources may still carry potentially neurotoxic chemical contaminants, creating a false sense of safety. Water quality assessment, especially in high-risk geological settings, needs to be assessed in both microbial and chemical terms. Remarkably, integrated approaches focusing on WASH and nutrition have yielded significant growth improvements in children, unlike single-sector interventions. Providing safe water and sanitation or implementing household water treatment, for example, does little to reduce fecal exposure pathways and consequently does little to improve growth in young children. Randomized control trials show that water quality interventions do not improve child growth, despite reductions in water contamination [26,27]. Child exposure to fecal contamination occurs through many pathways, including hand–mouth contamination (via touch, soil or animal feces), food intake (either through contaminated complementary foods or direct geophagy), and animal feces in the child’s environment, leaving them vulnerable to enteric pathogens. Focusing on one route, such as quality of water, does little to change the overall situation. Integrated WASH-nutrition interventions improve sanitation, handwashing, safe food preparation, and optimal feeding. These interventions address more pathways and mitigate the absence of adequate nutrition, exposing children to fewer pathogens overall. This explains the success of such approaches in comparison to single-sector interventions [13,24,33].

### 4.5. Policy Implications and Recommendations

The systematic review includes findings that have significant implications for policy and programming in low- and middle-income countries that differ based on the respective settings. From the synthesis of evidence provided, we delineate divergent policy approaches for varying contexts. In settings of moderate burden, similar to those of Cameron et al. [29] and Johri et al. [28], integrated water, sanitation, and hygiene (WASH) programs together with nutrition interventions show much higher effectiveness than single-sector interventions. This evidence comes from studies of combined WASH-nutrition approaches that yield significant improvements in child growth in instances where other interventions, such as water treatment or sanitation, did not lead to child growth improvements. Johri et al. documented that improved sources that are also E. coli-free, termed safely managed water, reduced underweight by 7.4% (95% CI: −13.4% to −1.3%) in rural India, though effects on stunting were not significant. This suggests that in contexts of moderate burden, water quality is one of several limiting factors for child growth, and simultaneous interventions on nutrition, sanitation, and water quality are crucial for meaningful improvements [22,27,33].

Nevertheless, the situation changes in extreme-burden settings with baseline stunting higher than 35% and water contamination rates exceeding 70% (the contexts of the null randomized controlled trials [21,26,27]), where household-level interventions seem inadequate. These settings are likely to require system-level changes, including piped water, sewered sanitation, and complete systems of integrated environmental sanitation (the investments and commitments are beyond what system changes entail). In such contexts, household interventions and point-of-use treatment are unlikely to make a difference. While such infrastructural investments are likely to take a long time, the null trials show that household interventions are likely to be a waste of time if the goal is to achieve significant improvements in child health. This highlights the importance of closing gaps to achieve SDG 6. Ensuring access to safely managed drinking water and sanitation is critical for child health and development [34].

The results of our review have culminated in several distinct priorities. First, water quality monitoring must expand to include monitoring quality at the point of consumption. There continues to be a major gap between the improved water source and actually safe water [32]. Surveillance systems must be strengthened to capture both microbial and chemical aspects of water quality, especially for emerging contaminants in higher-risk geologies [35]. This is evident in Pakhtigian’s study of negative effects in India due to water access, likely due to arsenic or fluoride [30].

Second, community-led interventions, like Community-Led Total Sanitation (CLTS), are scalable in high-burden contexts and have the potential for enduring shifts in behavior [36]. In the Pickering et al. study in Mali [22], community-level interventions that focused on wide-ranging environmental contamination were able to address child growth in a way that household-level interventions had not. Third, prioritizing water quality during the first 1000 days of life and pregnancy is essential, as the impact on growth and cognitive development is most severe during this period [37,38]. The long-term effects of early life exposures are highlighted in Wulan et al.’s study [21], which found that prenatal water quality predicted cognitive outcomes at ages 9−12 years.

Lastly, the long-term cognitive and economic benefits of water quality improvements during early childhood, as well as the cost-effectiveness of various intervention strategies across different contexts, have yet to be investigated [39,40]. The economic consequences of low-quality water, as analyzed by Cameron et al. [29] and Pakhtigian [30], are likely to go beyond immediate health-related costs and include partial lifetime earnings due to lower productivity as a consequence of cognition-impacting deficiencies. However, the current literature is quiet regarding these long-term consequences.

### 4.6. Monitoring Beyond Access Metrics

As noted in many studies we reviewed, including Lauer et al. [24], the current SDG 6 indicators focus on the accessibility of “improved” water sources, failing to address water quality at the point of use and related health outcomes. Contamination can occur during collection, transport, storage, and use, and the focus on water source rather than on water quality at the point of use ignores critical aspects of the water use continuum. This highlights the importance of the difference between “improved” sources and actual microbial or chemical safety. Similarly, Pakhtigian’s findings, while not excluding improved sources in India, noted negative outcomes resulting from chemical contamination [30].

We encourage dual-use monitoring frameworks that would enable empirical linkage of water quality and child health outcomes. One way this might be accomplished is by combining point-of-use water quality testing with existing national surveys (e.g., demographic and health surveys) that include child weight and height measurements. This would allow for the assessment of water quality and health impacts at the population level and would serve to substantiate the equitable distribution of resources based on evidence, while preserving the ability to compare countries and years.

The limitations must be acknowledged. Analyze the evidence surrounding cognitive predictors with appropriate caution. Only three studies in this review focused on cognitive development, using diverse assessment techniques across different age groups. Although there are studies indicating the relationship between the quality of water and cognitive function, the number of studies, small effect sizes, and methodological differentials categorize these findings as preliminary. Additional studies are needed with standardized cognitive assessment tools, larger populations, and extended follow-up periods in order to draw stronger conclusions about the impact of water quality on neurodevelopmental outcomes.

Although the majority of studies taking place in LMICs are highly relevant to the global burden of child undernutrition, they limit generalizability to high-income settings with different contaminant profiles, such as heavy metals or pharmaceuticals. Furthermore, the focus on anthropometric and cognitive outcomes largely ignores other potential health impacts, such as immune function or morbidity from infectious diseases, that may be associated with stunted growth and development and that may be interrelated or span across multiple domains.

Forthcoming studies should address the foremost questions that require attention. First, the impact of system-level infrastructure changes on child health will be evaluated using innovative study designs, such as the stepped-wedge approach, as infrastructure develops, given that large-scale randomization of infrastructure is impossible. Second, the economic and developmental ramifications of early-life water quality exposure will require longitudinal cognitive follow-ups extending into mid- and late adolescence. Third, the absence of systematic assessments of chemical contamination is especially acute and needs prioritization in areas of high-risk geology. Finally, cross-research with the aim of examining the variables that affect uptake, adherence, and sustainability of proven efficient interventions in resource-poor settings will advance the synthesis of evidence and practice.

## 5. Conclusions

The poor quality of drinking water, especially for children aged 0–5 years, affects growth and cognitive development due to microbiological (sp. *E. coli*) contamination and subsequent environmental enteric dysfunction (EED). Although water quality interventions appear to have mediocre results, combined WASH and nutrition strategies appear to have potential to reduce stunting and improve cognitive performance. Most evidence comes from low- and middle-income countries and specific WASH infrastructure; therefore, caution is warranted regarding the extrapolation of results to other countries lacking WASH that have different baseline sanitation and intervention implementation contexts. These results demonstrate the necessity for focused interventions to accomplish the SDG 6 targets and to improve child health in low- and middle-income countries.

## Figures and Tables

**Table 1:** Database search strategies.

Databases	Search Strategies	Records Found	Records Used
Scopus	“water quality” [MeSH Terms] OR (“child growth” [All Fields] AND “anthropometric outcomes” [All Fields])	161	11
EBSCO	“water quality” [MeSH Terms] OR (“child growth” [All Fields] AND “anthropometric outcomes” [All Fields])	7	0
PubMed	“water quality” [MeSH Terms] OR (“child growth” [All Fields] AND “anthropometric outcomes” [All Fields])	10	0
Cochrane	“water quality” [MeSH Terms] OR (“child growth” [All Fields] AND “anthropometric outcomes” [All Fields])	2	0
Google Search	“water quality” [MeSH Terms] OR (“child growth” [All Fields] AND “anthropometric outcomes” [All Fields])	42	4

**Table 2:** Descriptive summary of included studies on the impact of drinking water quality on child growth and development.

No.	Author (Year)	Study Design	Measurements	Main Findings	Conclusion, Strengths, and Limitations
1.	Dangour et al. (2013) [11]	Design: Meta-analysis including 14 studies (5 cluster randomized controlled trials (RCTs) and 9 non-randomized studies). Sample size: 22,241 children (<5 years) across 10 low- and middle-income countries (LMICs). Inclusion criteria: Studies assessing the impact of water, sanitation, and hygiene (WASH) interventions on child growth. Location: Multiple LMICs. Duration: 6–60 months.	Interventions included water quality (solar disinfection, bleach, filters), sanitation (latrines, toilets), hygiene (handwashing with soap), and combined WASH interventions.	Meta-analysis of 5 cluster RCTs (n = 4627) indicated small but significant improvements in height-for-age Z-scores (HAZ) (mean difference (MD) = 0.08; 95% confidence interval (CI): 0.00–0.16). No effect was found for weight-for-age or weight-for-height indices. Greater effects were observed among girls and children aged <24 months.	Some WASH interventions modestly improved child growth. Strengths: Inclusion of multiple countries and RCTs. Limitations: Low study quality, lack of masking, limited adherence and follow-up duration (9–12 months).
2.	Clasen et al. (2014) [20]	Design: Cluster RCT. Sample size: 9480 households (50 intervention, 50 control) across 100 villages. Inclusion criteria: Households with children <4 years or pregnant women. Location: Rural Odisha, India. Duration: 2010–2013 (18 months follow-up).	Intervention involved community sanitation (latrine construction). Outcomes included diarrhea prevalence, soil-transmitted helminth infection, anthropometry (weight, height), and environmental contamination.	Latrine coverage increased (9% → 63%), but no significant effects on diarrhea (8.80% vs. 9.10%), helminth infection, or child growth were found.	Sanitation intervention failed to improve health outcomes. Strengths: Large randomized design. Limitations: Limited latrine use, inability to mask intervention, short follow-up, self-reported outcomes.
3.	Wulan et al. (2015) [21]	Design: Longitudinal follow-up from the supplementation with multiple micronutrients intervention trial (SUMMIT). Sample size: 3687 children aged 9–12 years. Inclusion criteria: Children born to mothers in SUMMIT with baseline data on water, sanitation, and maternal education. Location: Indonesia.	Baseline data collected on household access to safe water and sanitation during pregnancy. Cognitive performance assessed using the Digit Span Forward, Information, Block Design, and Word List Memory tests. Adjusted for maternal education, socioeconomic status, and home environment.	Children whose mothers had access to safe water and toilets during pregnancy scored higher in cognitive tests (Word List Memory 95% CI: 0.01–0.19, *p* < 0.02).	Access to safe water and sanitation during pregnancy is linked to better child cognitive performance. Strengths: Large sample, longitudinal design. Limitations: Potential unmeasured confounding factors.
4.	Pickering et al. (2015) [22]	Design: Cluster RCT. Sample size: 4532 households in 121 villages (60 intervention, 61 control). Inclusion criteria: Villages eligible for community-led total sanitation (CLTS) program. Location: Koulikoro Region, Mali. Duration: 24 months (18-month post-intervention).	CLTS intervention aimed to eliminate open defecation through community mobilization. Outcomes included sanitation coverage, diarrhea prevalence, and child growth.	Sanitation coverage increased from 33% to 65%; open defecation decreased by 23% (adults) and 43% (children). Height-for-age Z-scores increased by 0.18; stunting reduced by 6 percentage points.	CLTS improved sanitation and child growth without financial subsidies. Strengths: Large RCT, behavioral focus. Limitations: Self-reported outcomes, no parasite testing, single-season follow-up.
5.	Freeman et al. (2017) [14]	Design: Systematic review and meta-analysis. Sample size: 171 studies (64 new since prior review). Inclusion criteria: Studies assessing sanitation impacts on health outcomes. Location: Global. Duration: Review period up to 2015.	Meta-analysis using random-effects models to estimate pooled effects of sanitation on diarrhea, trachoma, soil-transmitted helminths, schistosomiasis, and anthropometry. Quality assessed using the Liverpool quality appraisal tool (LQAT) and the grading of recommendations, assessment, development, and evaluation (GRADE) system.	Sanitation showed protective effects against diarrhea, trachoma, helminths, schistosomiasis, and HAZ, but not for other anthropometric outcomes. Evidence quality ranged from very low to high.	Sanitation improves select health outcomes. Strengths: Comprehensive synthesis. Limitations: Heterogeneity and low quality in many included studies.
6.	Hasanah et al. (2018) [23]	Design: Cross-sectional secondary analysis Sample size: 2835 children aged 0–5 years. Inclusion criteria: Children with complete stunting data. Location: 13 Indonesian provinces (representing 83% of the national population).	Exposure variables: water source (protected *vs*. unprotected) and sanitation type (proper *vs*. improper). Outcomes: stunting (HAZ < -2 SD), anthropometry, parental and socioeconomic variables.	Proper sanitation reduced stunting likelihood (odds ratio (OR) = 0.645, *p* < 0.01). Protected water reduced stunting (OR = 0.87), but not significantly.	Proper sanitation significantly reduces stunting risk. Strengths: Large, representative sample. Limitations: Cross-sectional design; no direct water testing.
7.	Lauer et al. (2018) [24]	Design: Cross-sectional sub-study within the Uganda birth cohort study (UBCS). Sample size: 385 children aged 12–16 months. Inclusion criteria: Children with complete visits and no recent diarrhea or severe illness. Location: Rural southwestern Uganda.	Water quality measured using *E. coli* via the compartment bag test (CBT). Environmental enteric dysfunction (EED) assessed using lactulose:mannitol (L:M) ratio. Anthropometric indicators included length-for-age Z-score (LAZ), WAZ, and stunting.	Unsafe water associated with higher EED (β = −0.22, 95% CI: −0.44–0.00) and lower LAZ (β = 0.29, 95% CI: 0.00–0.58). Stunting odds 1.68–1.70 times higher in unsafe water groups.	Unsafe drinking water increases risk of EED and impaired growth. Strengths: Objective testing of water and biomarkers. Limitations: Single-time water testing, limited causal inference.
8.	Haque et al. (2019) [25]	Design: Cross-sectional analysis using the Bangladesh multiple indicator cluster survey (MICS) 2012–2013. Sample size: 816 children (household level) and 1292 (source level). Inclusion criteria: Children aged <5 years with complete height-for-age and *Escherichia coli* data. Location: Bangladesh.	Water contamination measured at household and source points using microbial tests (<1, 0–10, 11–200 colony-forming units (CFU)/100 mL). Outcomes: stunting (height-for-age Z-score (HAZ) < −2 standard deviations (SD)) and related WASH factors.	High *E. coli* contamination increased stunting risk by 9% at source and 6% at household points (*p* < 0.05). Associations stronger among children aged 24–60 months.	Poor water quality significantly increases stunting risk. Strengths: Nationally representative data, robust controls. Limitations: Cross-sectional design, single-time water sampling.
9.	Null et al. (2019) [26]	Design: Cluster randomized controlled trial (RCT). Sample size: 8246 pregnant women (6659 children followed up). Inclusion criteria: Pregnant women in rural Kenya; households without ongoing water, sanitation, and nutrition programs. Location: Rural Kenya.	Interventions: water chlorination, latrine upgrades, handwashing stations, and nutrition supplementation. Primary outcomes: caregiver-reported diarrhea and length-for-age Z-score (LAZ) at year 2.	Nutrition and combined WASH with nutrition groups improved LAZ (+0.13–0.16, *p* < 0.05); no improvements from water, sanitation, or handwashing alone. High baseline sanitation coverage (>70%) may have limited incremental effects.	WASH-only interventions did not improve child growth, while nutrition interventions had measurable benefits. Strengths: Large sample size, robust randomization, multi-arm design. Limitations: Declining adherence to water and hygiene components, absence of masking, and residual environmental contamination (e.g., animal feces, protozoa).
10.	Luby et al. 2019 [27]	Design: Cluster RCT. Sample size: 5551 pregnant women (4667 children followed up). Inclusion criteria: Rural households without existing WASH or nutrition programs; low groundwater iron/arsenic levels. Location: Bangladesh.	Interventions: chlorinated water, improved sanitation, handwashing, and lipid-based nutrient supplements (LNS). Primary outcomes: diarrhea and length-for-age Z-score (LAZ).	Diarrhea decreased in sanitation (3.5%) and handwashing (3.5%) groups; LAZ improved in nutrition (+0.25) and combined WASH + nutrition (+0.13) groups (95% CI: 0.01–0.36). Water-only interventions had no effect.	Nutrition-focused interventions significantly improved linear growth; WASH reduced diarrhea but not growth. Strengths: High adherence and low attrition rates. Limitations: Low baseline diarrhea prevalence, limited community-level sanitation impact, and lack of blinding.
11.	Johri et al. (2019) [28]	Design: Cross-sectional study using propensity score matching. Sample size: 1088 households with children aged 12–23 months. Inclusion criteria: Rural households in Uttar Pradesh, India. Location: Hardoi District, India.	Intervention: comparison of “safely managed” drinking water (improved source and E. coli-free) vs. “basic” water. Outcomes: stunting, underweight, and wasting (Z-scores < −2 SD).	Safely managed water reduced underweight by 7.4% (95% CI: −13.4% to −1.3%, *p* = 0.017), but effects on stunting and wasting were nonsignificant.	Safe water reduces underweight but not stunting in high-pathogen settings. Strengths: Microbial data, robust analysis. Limitations: Observational design, potential misclassification bias.
12.	Cameron et al. (2020) [29]	Design: Longitudinal analysis using Indonesia Family Life Survey (IFLS) 1993–2014. Sample size: 6365 children (stunting analysis), 4150 (cognitive outcomes). Inclusion criteria: Children aged 0–59 months with complete WASH and outcome data. Location: Indonesia	WASH exposures (household sanitation, water access, community open defecation rates) linked with anthropometric and cognitive outcomes. Cognitive tests assessed language, memory, and numeracy.	Improved sanitation reduced stunting by 4–5% and increased HAZ by 0.19–0.27 SD (*p* < 0.01). Open defecation–free (ODF) communities increased cognitive scores by 4.5–7.5% (*p* < 0.05).	Sanitation had stronger effects on growth and cognition than water access. Strengths: Large longitudinal dataset. Limitations: Observational design; limited direct water testing.
13.	Pakhtigian (2024) [30]	Design: Longitudinal panel analysis using Young Lives data. Sample size: 8062 children across Ethiopia, India, Peru, and Vietnam. Inclusion criteria: Households with early-life WASH data and follow-up to adolescence. Location: Multi-country (Ethiopia, India, Peru, Vietnam).	Early-life access to improved drinking water and sanitation assessed using World Health Organization/UNICEF Joint Monitoring Programme (WHO/UNICEF JMP) definitions. Cognitive tests (math, reading, Peabody Picture Vocabulary Test (PPVT)) administered at age ~15 years.	Improved water access in early life increased math (+2–4%), reading (+3–5%), and PPVT (+2–3%) scores (*p* < 0.05), especially among girls. Negative results in India may reflect fluoride contamination.	Early access to safe water enhances adolescent cognition. Strengths: Long-term, multi-country data. Limitations: Correlational analysis; limited contaminant testing.
14.	Syaputri et al. (2023) [31]	Design: Case–control study. Sample size: 84 children (42 stunted, 42 non-stunted) aged 6–59 months. Inclusion criteria: Children attending Talun Kenas Health Center. Location: Deli Serdang Regency, Indonesia.	Water quality assessed physically, chemically, and microbiologically following Indonesian Health Regulation. Outcomes: stunting (HAZ < −2 SD).	Poor water quality increased stunting risk 4.14-fold (OR = 4.144, 95% CI: 1.646–10.435, *p* = 0.004).	Poor water quality significantly increases stunting risk. Strengths: Direct water testing. Limitations: Small sample size; single-location study.
15.	Som et al. (2023) [32]	Design: Cross-sectional secondary analysis Sample size: 3375 children aged 6–24 months (weighted n = 3345). The Lao social indicator survey II (LSIS II, 2017). Inclusion criteria: Children with complete anthropometric data and water quality testing. Location: Lao People’s Democratic Republic (Lao PDR).	Exposures: WASH indicators, and infant and young child feeding (IYCF) practices. Water quality: *E. coli* contamination testing of household and source water. Outcomes: Stunting (LAZ < –2 SD) and wasting (WLZ < –2 SD).	Combined WASH with IYCF interventions reduced stunting (AOR = 0.54, 95% CI: 0.41–0.73) and wasting (AOR = 0.64, 95% CI: 0.44–0.92). Improved water sources alone had no effect due to 86.5% contamination of household water and 83.6% of sources with *E. coli*.	Integrated WASH and nutrition interventions were more effective than single interventions in reducing undernutrition. Strengths: Nationally representative dataset, inclusion of microbial testing. Limitations: Cross-sectional design and limited behavioral/hygiene data.

## Data Availability

Data sharing is not applicable to this article as no new datasets were generated or analyzed during the current study.

## Supplementary Materials

The following supporting information can be downloaded at: https://www.mdpi.com/article/10.3390/ijerph23030313/s1, File S1: PRISMA Checklist.

## Abbreviations

The following abbreviations are used in this manuscript:

AOR	Adjusted Odds Ratio
CBT	Compartment Bag Test
CFU	Colony-Forming Units
CI	Confidence Interval
CLTS	Community-Led Total Sanitation
E. coli	Escherichia coli
EBSCO	Elton B. Stephens Company
EED	Environmental Enteric Dysfunction
GRADE	Grading of Recommendations, Assessment, Development, and Evaluation
HAZ	Height-for-Age Z-score
IC1	Inclusion Criterion 1
IC2	Inclusion Criterion 2
IFLS	Indonesia Family Life Survey
IYCF	Infant and Young Child Feeding
JMP	Joint Monitoring Programme (WHO/UNICEF)
LAZ	Length-for-Age Z-score
LMICs	Low- and Middle-Income Countries
LNS	Lipid-Based Nutrient Supplements
LQAT	Liverpool Quality Appraisal Tool
MD	Mean Difference
MeSH	Medical Subject Headings
MICS	Multiple Indicator Cluster Survey
NOS	Newcastle–Ottawa Scale
ODF	Open Defecation-Free
OR	Odds Ratio
PPVT	Peabody Picture Vocabulary Test
PRISMA	Preferred Reporting Items for Systematic Reviews and Meta-Analyses
PRISMA-S	PRISMA Extension for Reporting Literature Searches
RCT	Randomized Controlled Trial
SD	Standard Deviation
SDG	Sustainable Development Goal
SUMMIT	Supplementation with Multiple Micronutrients Intervention Trial
UBCS	Uganda Birth Cohort Study
UNICEF	United Nations Children’s Fund
WASH	Water, Sanitation, and Hygiene
WAZ	Weight-for-Age Z-score
WLZ	Weight-for-Length Z-score
WHO	World Health Organization
