# Supplementary material for: Safe Drinking Water and Its Impact on Children’s Growth and Development: A Systematic Review
Source: Int J Environ Res Public Health. 2026 Mar 2;23(3):313. doi: 10.3390/ijerph23030313 (PMC13027215; doi:10.3390/ijerph23030313)
Supplement: Supplementary file 1 [file ijerph-23-00313-s001.zip › ijerph-4075918-supplementary.pdf]

PRISMA

PRISMA 2020 Checklist

Section and Topic	Item #	Checklist item	Location where item is reported
**TITLE**			
Title	1	Identify the report as a systematic review.	Title page: Safe Drinking Water and Its Impact on Children's Growth and Development: A Systematic Review
**ABSTRACT**			
Abstract	2	See the PRISMA 2020 for Abstracts checklist.	Abstract section (page 2): Structured abstract includes background, methods (search strategy, databases, inclusion criteria), results (15 studies included), and conclusions.
**INTRODUCTION**			
Rationale	3	Describe the rationale for the review in the context of existing knowledge.	Introduction section (pages 2-3): Discusses the global burden of contaminated drinking water, environmental enteric dysfunction (EED), stunting prevalence, and the gap in systematic reviews focusing specifically on drinking water quality.
Objectives	4	Provide an explicit statement of the objective(s) or question(s) the review addresses.	Introduction section (page 3): This systematic review aims to synthesize evidence from prospective cohort studies and clinical trials to evaluate the impact of drinking water quality on child growth and development, focusing on anthropometric outcomes
**METHODS**			
Eligibility criteria	5	Specify the inclusion and exclusion criteria for the review and how studies were grouped for the syntheses.	Section 2.2 Eligibility Criteria (pages 4-5): Two sets of inclusion criteria (IC1 and IC2) clearly defined. IC1: peer-reviewed English articles 2010-2025. IC2: children 0-5 years, prospective cohort/RCTs, quantitative measures of growth/development. Exclusion criteria also specified.
Information sources	6	Specify all databases, registers, websites, organisations, reference lists and other sources searched or consulted to identify studies. Specify the date when each source was last searched or consulted.	Section 2.3 Information Sources and Search Strategy (page 5) and Table 1: Five databases searched (SCOPUS, EBSCO, PubMed, Cochrane Library, Google Search). Date range: January 2010 to May 2025. Reference lists manually reviewed.
Search strategy	7	Present the full search strategies for all databases, registers and websites, including any filters and limits used.	Section 2.3 and Table 1 (page 5): Search terms listed including
Selection process	8	Specify the methods used to decide whether a study met the inclusion criteria of the review, including how many reviewers screened each record and each report retrieved, whether they worked independently, and if applicable, details of automation tools used in the process.	Section 2.4 Study Selection Process (page 5) and Figure 1 (page 4): Four-phase selection per Rethlefsen et al. Authors screened independently, disagreements resolved through discussion. No automation tools used.
Data collection process	9	Specify the methods used to collect data from reports, including how many reviewers collected data from each report, whether they worked independently, any processes for obtaining or confirming data from study investigators, and if applicable, details of automation tools used in the process.	Section 2.5 Data Collection and Quality Assessment (page 6): Standardized extraction form used. Authors worked independently for data extraction and quality assessment, with discrepancies resolved through discussion.
Data items	10a	List and define all outcomes for which data were sought. Specify whether all results that were compatible with each outcome domain in each study were sought (e.g. for all measures, time points, analyses), and if not, the methods used to decide which results to collect.	Section 2.5 (page 6): Outcomes extracted included study design, sample size, demographics, drinking water quality, anthropometric outcomes (stunting, HAZ, WAZ), and cognitive outcomes (memory, language, numeracy test scores) with effect sizes and confidence intervals.
	10b	List and define all other variables for which data were sought (e.g. participant and intervention characteristics, funding sources). Describe any assumptions made about any missing or unclear information.	Section 2.5 and Table 2: Variables extracted include participant characteristics (age, location), intervention details, water quality measurements (E. coli, total coliform), sample sizes, and study conclusions.

Section and Topic	Item #	Checklist item	Location where item is reported
			Heterogeneity noted.
Study risk of bias assessment	11	Specify the methods used to assess risk of bias in the included studies, including details of the tool(s) used, how many reviewers assessed each study and whether they worked independently, and if applicable, details of automation tools used in the process.	Section 2.5 (page 6) and Section 3.3: Newcastle-Ottawa Scale (NOS) used for cohort studies and Cochrane risk of bias tool for clinical trials. Authors worked independently with disagreements resolved through discussion.
Effect measures	12	Specify for each outcome the effect measure(s) (e.g. risk ratio, mean difference) used in the synthesis or presentation of results.	Results section (pages 9-10): Effect measures reported include odds ratios (OR) with 95% CI for stunting, beta coefficients for HAZ scores, mean differences for cognitive scores, and percentage changes.
Synthesis methods	13a	Describe the processes used to decide which studies were eligible for each synthesis (e.g. tabulating the study intervention characteristics and comparing against the planned groups for each synthesis (item #5)).	Section 2.5 (page 6): Studies categorized by outcome type (anthropometric vs cognitive). Heterogeneity in study designs, water quality measurements, and outcomes precluded meta-analysis; narrative synthesis conducted.
	13b	Describe any methods required to prepare the data for presentation or synthesis, such as handling of missing summary statistics, or data conversions.	Section 2.5 (page 6): Standardized extraction form used. Section 3.3 notes heterogeneity considerations.
	13c	Describe any methods used to tabulate or visually display results of individual studies and syntheses.	Figure 1 (PRISMA flow diagram) on page 4 and Table 2 (descriptive summary of included studies) on pages 6-9.
	13d	Describe any methods used to synthesize results and provide a rationale for the choice(s). If meta-analysis was performed, describe the model(s), method(s) to identify the presence and extent of statistical heterogeneity, and software package(s) used.	Section 2.5 (page 6): Narrative synthesis employed due to substantial heterogeneity in study designs, water quality measurements (E. coli, total coliform), outcomes (stunting cut-offs, cognitive assessments), and populations. No meta-analysis performed.
	13e	Describe any methods used to explore possible causes of heterogeneity among study results (e.g. subgroup analysis, meta-regression).	Section 3.3 (page 10): Heterogeneity attributed to different study environments, contaminant types (microbial vs chemical), and outcome measurements. No formal subgroup analysis or meta-regression conducted.
	13f	Describe any sensitivity analyses conducted to assess robustness of the synthesized results.	Not applicable - No sensitivity analyses conducted due to narrative synthesis approach and heterogeneity precluding quantitative pooling.
Reporting bias assessment	14	Describe any methods used to assess risk of bias due to missing results in a synthesis (arising from reporting biases).	Section 3.3 (page 10): Study quality limitations discussed including lack of blinding, variable intervention adherence, and use of proxy measures. No formal reporting bias assessment conducted.
Certainty assessment	15	Describe any methods used to assess certainty (or confidence) in the body of evidence for an outcome.	Section 3.3 (page 10): Quality ratings (mostly moderate) using NOS and Cochrane tools. Limitations noted including measurement heterogeneity and lack of blinding.
**RESULTS**			
Study selection	16a	Describe the results of the search and selection process, from the number of records identified in the search to the number of studies included in the review, ideally using a flow diagram.	Section 2.4 (pages 5) and Figure 1: 222 records identified, 207 excluded after title/abstract screening, 15 studies included in final qualitative synthesis. PRISMA flow diagram provided.
	16b	Cite studies that might appear to meet the inclusion criteria, but which were excluded, and explain why they were excluded.	Section 2.4 (page 5): 207 records removed due to irrelevance, duplication, or failure to satisfy inclusion criteria IC1. Reference list screening found additional studies noncompliant with inclusion criteria.
Study characteristics	17	Cite each included study and present its characteristics.	Table 2 (pages 6-9): Comprehensive descriptive summary of all 15 included studies with author, year, study design, sample size, measurements, main findings, and conclusions. References 8, 20-34 in References section.

PRISMA 2020 Checklist

Section and Topic	Item #	Checklist item	Location where item is reported
Risk of bias in studies	18	Present assessments of risk of bias for each included study.	Section 3.3 (page 10): Quality assessment using NOS for cohort studies and Cochrane tool for clinical trials. Most studies rated moderate quality with noted limitations (lack of blinding, variable adherence, proxy measures).
Results of individual studies	19	For all outcomes, present, for each study: (a) summary statistics for each group (where appropriate) and (b) an effect estimate and its precision (e.g. confidence/credible interval), ideally using structured tables or plots.	Table 2 (pages 6-9): Main findings column presents effect estimates with 95% CI, odds ratios, beta coefficients, mean differences, and p-values for each study.
Results of syntheses	20a	For each synthesis, briefly summarise the characteristics and risk of bias among contributing studies.	Sections 3.1 and 3.2 (page 9) and Section 3.3 (page 10): Narrative synthesis by outcome type. Anthropometric outcomes (10 studies, 8 found associations). Cognitive outcomes (5 studies, 3 found associations). Quality assessment in Section 3.3.
	20b	Present results of all statistical syntheses conducted. If meta-analysis was done, present for each the summary estimate and its precision (e.g. confidence/credible interval) and measures of statistical heterogeneity. If comparing groups, describe the direction of the effect.	Sections 3.1 and 3.2 (pages 9-10): Narrative synthesis presented. No meta-analysis conducted due to heterogeneity. Direction of effects described (increased stunting risk, reduced HAZ, improved cognitive scores with safe water).
	20c	Present results of all investigations of possible causes of heterogeneity among study results.	Section 3.3 (page 10): Heterogeneity attributed to different study environments, contaminant types (microbial vs chemical), and outcome measurements. No formal heterogeneity investigation conducted.
	20d	Present results of all sensitivity analyses conducted to assess the robustness of the synthesized results.	Not applicable - No sensitivity analyses conducted.
Reporting biases	21	Present assessments of risk of bias due to missing results (arising from reporting biases) for each synthesis assessed.	Section 3.3 (page 10): Study limitations discussed but no formal reporting bias assessment conducted.
Certainty of evidence	22	Present assessments of certainty (or confidence) in the body of evidence for each outcome assessed.	Section 3.3 (page 10): Most studies rated moderate quality using NOS and Cochrane tools. Limitations noted in evidence quality.
**DISCUSSION**			
Discussion	23a	Provide a general interpretation of the results in the context of other evidence.	Discussion section (pages 10-17): Results interpreted in context of EED mechanisms, global child malnutrition burden, WASH interventions, and SDG 6 targets. Compared with existing literature on WASH and child health.
	23b	Discuss any limitations of the evidence included in the review.	Discussion section (pages 10-17): Limitations discussed including heterogeneity in water quality measurements, reliance on proxy indicators (E. coli), variable intervention adherence, and limited data on chemical contaminants and cognitive outcomes.
	23c	Discuss any limitations of the review processes used.	Discussion section (pages 10-17): Review limitations include restriction to English-language publications, absence of formal meta-analysis due to heterogeneity, and lack of systematic review registration.
	23d	Discuss implications of the results for practice, policy, and future research.	Discussion section and Conclusions (pages 10-17): Implications for integrated WASH-nutrition programs, policy recommendations for SDG 6 implementation, need for standardized water quality measurements, and future research on chemical contaminants and cognitive outcomes.
**OTHER INFORMATION**			
Registration and protocol	24a	Provide registration information for the review, including register name and registration number, or state that the review was not registered.	Not applicable

PRISMA 2020 Checklist

Section and Topic	Item #	Checklist item	Location where item is reported
	24b	Indicate where the review protocol can be accessed, or state that a protocol was not prepared.	Not applicable
	24c	Describe and explain any amendments to information provided at registration or in the protocol.	Not applicable
Support	25	Describe sources of financial or non-financial support for the review, and the role of the funders or sponsors in the review.	Funding section (page 17): "This study was funded by PT Tirta Investama, Indonesia"
Competing interests	26	Declare any competing interests of review authors.	Conflicts of Interest section (page 17): "Author Tria Rosemiarti was employed by PT Tirta Investama, Indonesia. The remaining authors declare no conflicts of interest."
Availability of data, code and other materials	27	Report which of the following are publicly available and where they can be found: template data collection forms; data extracted from included studies; data used for all analyses; analytic code; any other materials used in the review.	Data Availability Statement section (page 17): Data sharing is not applicable to this article as no new datasets were generated or analyzed during the current study

From: Page MJ, McKenzie JE, Bossuyt PM, Boutron I, Hoffmann TC, Mulrow CD, et al. The PRISMA 2020 statement: an updated guideline for reporting systematic reviews. BMJ 2021;372:n71. doi: 10.1136/bmj.n71. This work is licensed under CC BY 4.0. To view a copy of this license, visit https://creativecommons.org/licenses/by/4.0/
